# Urinary bladder duplication in an adult male with associated genitourinary and spinal malformations: Multimodal imaging and surgical confirmation in a complex urogenital case

**DOI:** 10.1016/j.radcr.2025.09.077

**Published:** 2025-10-24

**Authors:** Priyanka R Prakash, Vipul K Rathan, Santosh Phajir Vishwanath Rai

**Affiliations:** Department of Radiodiagnosis, Kasturba Medical College Mangalore, Manipal Academy of Higher Education, Manipal, India

**Keywords:** Congenital urogenital malformations, Urinary bladder duplication, left renal hypoplasia, VUJ bladder diverticulum, Caudal regression, Hypoplastic prostate

## Abstract

Bladder duplication is a rare congenital anomaly, seldom reported in adults. We describe a young male presenting with recurrent urinary symptoms and lower abdominal pain. Imaging (ultrasound, CT urogram, MRI) revealed a complete sagittal septum dividing the bladder into 2 cavities with cystolithiasis, along with a hypoplastic left kidney, vesicoureteral junction (VUJ) diverticulum, partial sacral hypoplasia, and hypoplastic prostate and seminal vesicles. The patient underwent septum resection and stone removal, leading to symptom resolution. This case underscores the importance of considering congenital anomalies in adult urological presentations and highlights the role of comprehensive imaging in diagnosis and management.

## Introduction

Bladder duplication (BD) is an exceptionally rare congenital anomaly of the urogenital system, characterized by the presence of 2 anatomically distinct bladder cavities. Fewer than 100 cases have been documented worldwide, with a clear male predominance [1,2]. Although BD may remain asymptomatic and is often discovered incidentally—either during voiding cystourethrography or at autopsy—its identification warrants a comprehensive evaluation due to the high frequency of associated anomalies [3].

Up to 85% of BD cases are accompanied by reproductive system abnormalities, while 40%-56% present with colon duplication or anorectal malformations. In female patients, vaginal duplication is seen in approximately 90% of cases [4]. Other associated anomalies include septate uterus, uterine fibroids, urethral duplication with pelviureteric junction obstruction, renal or gonadal agenesis, non-functional kidneys, diphallus, congenital megacolon, imperforate anus, bowel obstruction, and hypospadias [4]. Spinal malformations are also observed in 10%-15% of patients [4].

The clinical presentation of BD is highly variable. While many individuals remain asymptomatic, others may experience recurrent urinary tract infections (UTIs), vesicoureteral reflux (VUR), cystolithiasis, urinary tract obstruction, persistent urogenital sinus, or even renal failure [5]. Given the risk of significant complications and the potential for delayed diagnosis, it is essential to recognize the imaging characteristics of BD across various modalities and to maintain a high index of suspicion for associated anomalies during patient evaluation.

## Case presentation

A young adult male presented with a long-standing history of lower abdominal discomfort, increased urinary frequency, and a sensation of incomplete bladder emptying. He also reported episodes of burning micturition and intermittent low-grade fever persisting over several years, partially alleviated by empirical medical treatment.

Notably, he had undergone surgical correction for an imperforate anus during infancy, suggesting a possible underlying complex congenital anomaly involving the caudal developmental field. However, no surgical records or prior imaging studies were available for review.

The patient denied any history of chronic medical conditions such as diabetes mellitus, hypertension, or tuberculosis. There was also no family history of congenital anomalies or chronic kidney disease. He recalled multiple episodes of urinary tract infections during childhood and early adulthood, which had been managed symptomatically without extensive evaluation or follow-up.

On examination, the patient was hemodynamically stable. Abdominal palpation revealed suprapubic tenderness, with no palpable organomegaly or flank fullness.

Laboratory investigations revealed a hemoglobin level of 131 g/L (reference: 130-170 g/L) and a total leukocyte count of 8.15 × 10⁹/L (reference: 4-11 × 10⁹/L). Blood urea was mildly elevated at 40 mg/dL (reference: 10-40 mg/dL), and serum creatinine was 1.6 mg/dL (reference: 0.6-1.2 mg/dL), indicating preserved yet potentially compromised renal function. Serum electrolytes were within normal limits. Both blood and urine cultures showed no microbial growth, and urine analysis was unremarkable.

Given the patient's symptoms and background, he was referred for abdominal ultrasonography to further evaluate his urinary tract and identify any structural abnormalities.

**Fig. 1:**
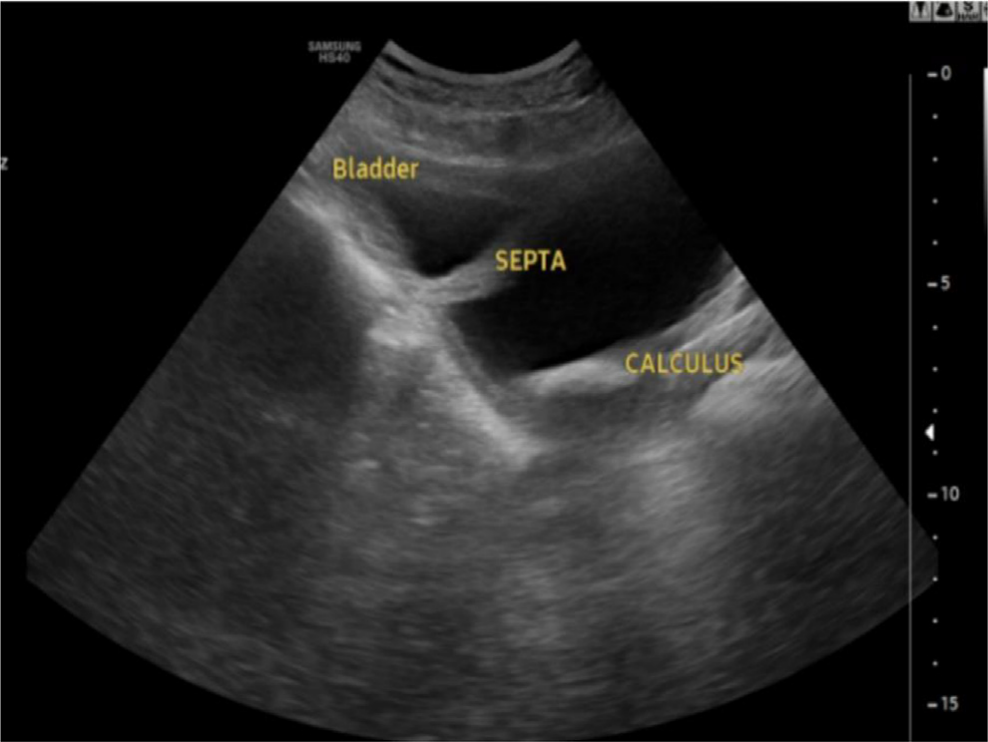
Abdominal ultrasound image (axial plane) showing thick hyperechoic septum dividing the bladder into 2 cavities with multiple free floating internal echoes and a calculus in left compartment of the bladder.

Abdominal ultrasonography revealed a distended urinary bladder, notably divided by a thick, hyperechoic sagittal septum into 2 distinct cavities. A mobile, dependent calculus was identified within the left bladder compartment (Fig. 1). The right kidney exhibited moderate hydroureteronephrosis, while the left kidney could not be visualized, raising concerns about renal agenesis or severe dysplasia. Additionally, the prostate and seminal vesicles were not clearly delineated on ultrasound. Based on these findings, a large left-sided bladder diverticulum was initially considered as a differential diagnosis.

**Fig. 2:**
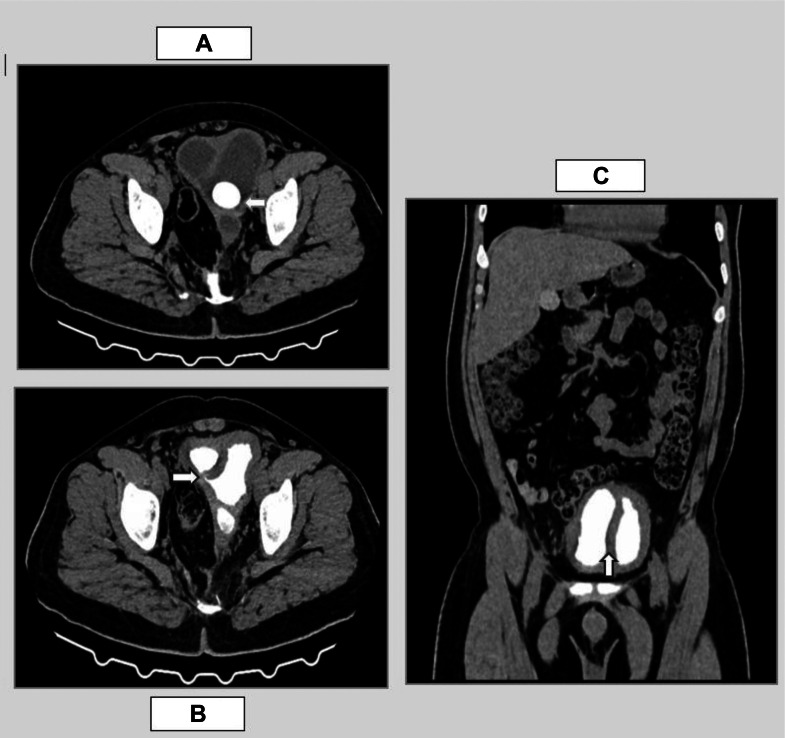
(A) Axial section of plain CT showing a calculus in left compartment of bladder (depicted by white arrow). (B) Axial section of CT urogram showing a small communication between the 2 bladder cavities at the neck of bladder (annotated by white arrow). (C) Coronal section of CT Urogram revealing a thick fibromuscular sagittal septum with attenuation similar to that of bladder wall (Annotated by white arrow) dividing the contrast filled urinary bladder into 2 separate compartments.

To further characterize the renal and urinary tract anatomy, a CT urogram was performed. This provided greater anatomical detail and clarified the nature of the septated bladder. The scan revealed a thick (∼8 mm) fibromuscular sagittal septum with attenuation characteristics similar to the bladder wall, effectively partitioning the contrast-opacified bladder into 2 compartments. A vesical calculus was confirmed within the left bladder cavity (Fig. 2A). The right compartment demonstrated normal drainage into the urethra, whereas the left compartment lacked urethral communication, suggesting functional obstruction. However, a small communication was identified between the 2 bladder cavities near the bladder neck (Fig. 2, Fig. 2), likely allowing partial decompression.

**Fig. 3:**
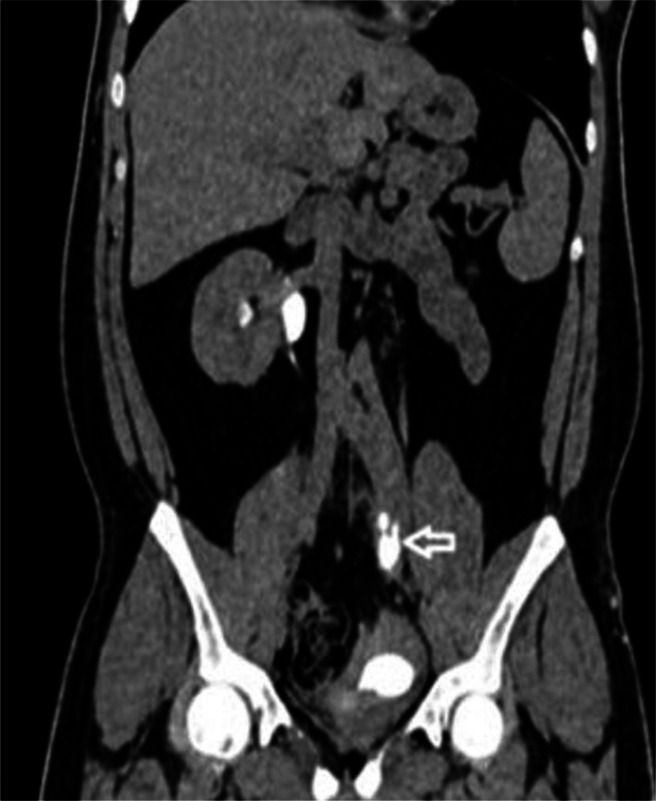
A coronal image of CT urogram depicting hypoplastic left kidney with remnants of left renal tissue showing normal contrast enhancement with delayed excretion into malformed primitive calyceal remnants (annotated by white arrow).

**Fig. 4:**
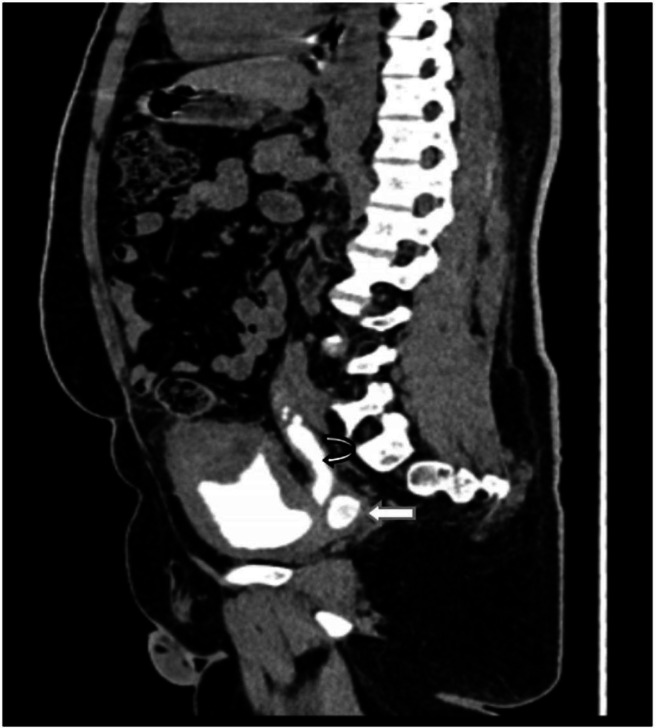
Image of excretory phase of CT urogram in sagittal plane showing left primitive ureter draining into left bladder compartment (annotated by curved white arrow) with a VUJ diverticulum at the draining point (depicted by white arrow).

Further evaluation of the upper urinary tract revealed that the right kidney was normal in size (∼11 × 6.6 cm) but showed moderate hydroureteronephrosis with mildly delayed contrast excretion. The right ureter was seen inserting into the right bladder compartment. In contrast, the left kidney was hypoplastic, with residual renal parenchyma measuring approximately 1.3 × 2.7 × 2.3 cm (AP × TR × CC), and showed significantly delayed contrast excretion (Fig. 3). The left ureter drained into the left bladder compartment, with a vesicoureteral junction (VUJ) diverticulum observed at its insertion site (Fig. 4). These CT findings, were consistent with a rare variant of complete BD, complicated by left-sided outflow obstruction and cystolithiasis, rather than a simple diverticulum.

Additional congenital anomalies were also identified, including hypoplastic seminal vesicles, hypoplastic prostate, and partial sacral agenesis, findings suggestive of Type II caudal regression syndrome [6].

**Fig. 5:**
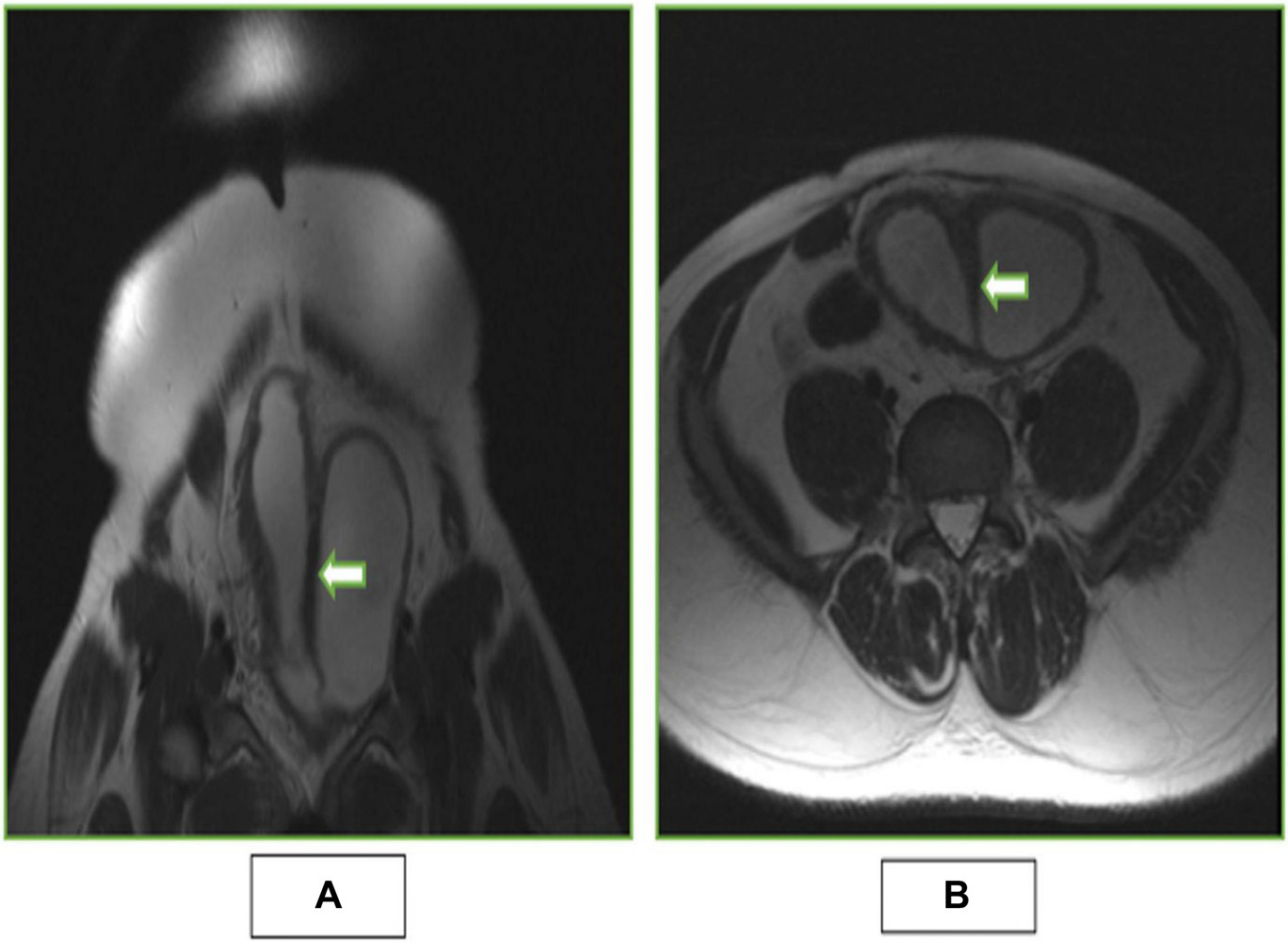
(A) Coronal T2 weighted image showing hypointense thick sagittal septum which was isointense to bladder wall (depicted by white arrow) separating the 2 bladder cavities with small communication between the compartments at neck of bladder (annotated by white arrow). (B) Axial T2 weighted image showing a thick sagittal septum isointense to bladder wall (depicted by white arrow) separating the 2 bladder cavities.

To further delineate the internal bladder anatomy, especially the septum, a limited MRI was performed. Axial T1- and T2-weighted images demonstrated a well-defined sagittal septum, hypointense on both sequences and isointense to the bladder wall, dividing the bladder into 2 cavities. A small intercommunication between the 2 compartments at the bladder neck was again noted, confirming prior CT findings **(**Figs. 5A and B).

Based on the combined imaging features, a diagnosis of a variant of complete sagittal BD with a small intercommunication at the bladder neck was established.

## Treatment

After a detailed interdepartmental discussion between the radiology and urology teams, a provisional diagnosis of urinary BD was established based on clinical presentation and multimodal imaging. Surgical intervention was advised to relieve symptoms, prevent further complications, and confirm the diagnosis intraoperatively.

The patient was thoroughly counselled about the condition and the proposed surgical plan. Following informed consent, he was taken up for surgery.

Intraoperative findings revealed a lobulated urinary bladder, divided into 2 distinct compartments by a thick fibromuscular sagittal septum. A small communication between the 2 cavities was identified at the bladder neck. The surgical team confirmed the diagnosis of complete BD.

**Fig. 6:**
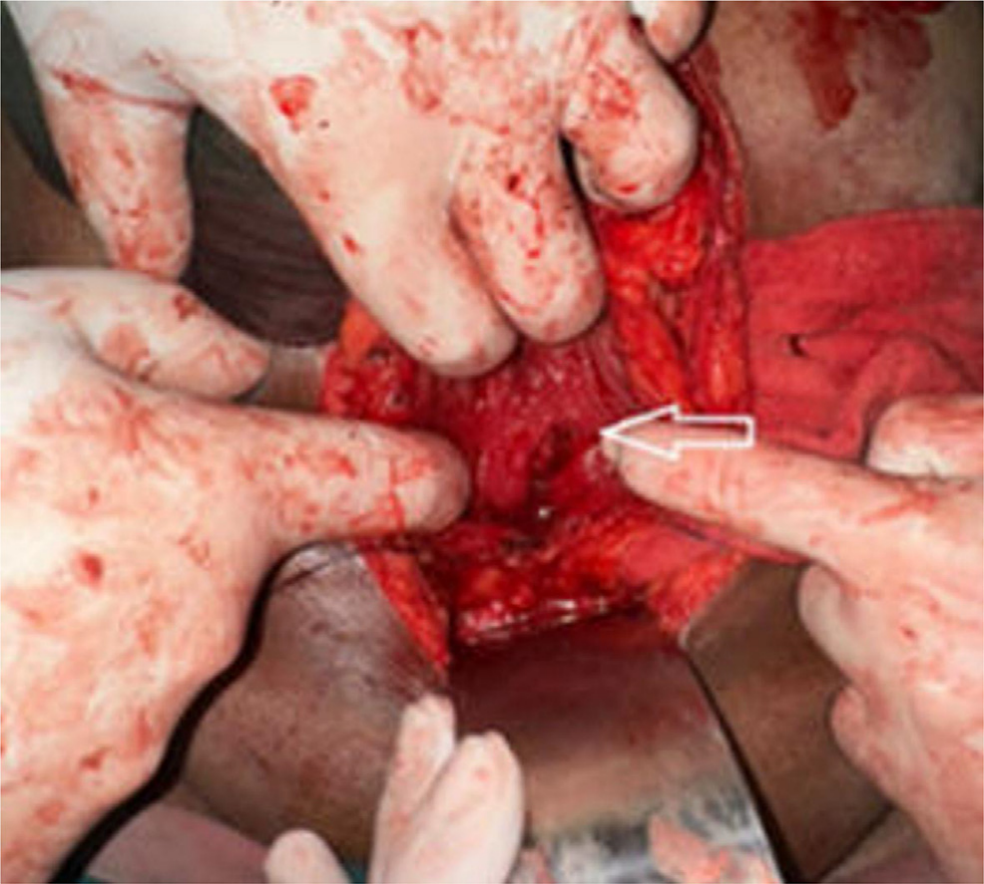
An intraoperative image demonstrating the partial resection of the septum to create a communication between the 2 bladder cavities (depicted by white arrow).

The septum was partially resected, and a wide communication was surgically created between the 2 bladder compartments to allow effective drainage (Fig. 6). A vesical calculus located in the left compartment was successfully extracted. To address the right-sided hydroureteronephrosis, a DJ stent was placed, and a suprapubic catheter was inserted for postoperative bladder decompression and healing.

## Outcome and follow-up

The patient tolerated the procedure well and remained hemodynamically stable throughout. The patient had an uneventful recovery and was discharged with instructions for follow-up after 6 weeks, or sooner if any urgent concerns developed. He was also advised to undergo a follow up CT urogram.

**Fig. 7:**
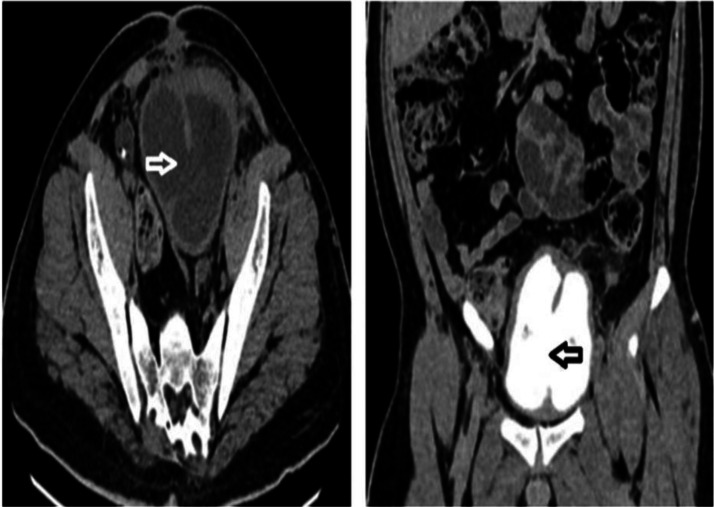
Follow-up CT urogram images: axial non contrast phase image demonstrating the post-operative resected septum, establishing communication between the bladder compartments (annotated by white arrow). Coronal delayed phase image demonstrating the post-operative resected septum (annotated by black arrow).

At the 6-week follow-up, the patient experienced resolution of symptoms, serum creatinine improved to 1.1 mg/dL, and he remained symptom-free at follow-up. CT urogram showed a partially resected septal defect (approximately 5.3 cm in craniocaudal length) with communication between bladder compartments (Fig. 7). The right kidney demonstrated normal contrast excretion with the DJ stent in situ. A follow-up appointment was scheduled in 6 months.

## Discussion

BD is an extremely rare congenital anomaly, with limited cases reported in the literature, particularly in adults. Most available data come from isolated case reports, making diagnosis and management challenging. Several published cases highlight this rarity and the diagnostic dilemmas posed. For instance, Dineva et al. [1] described a complex adult case of BD associated with disorder of sexual development, pancake kidney, and a neural tube defect, reinforcing the embryological association between urinary, genital, and spinal anomalies.

Luo et al. reported sagittal BD coexisting with posterior urethral duplication and congenital megacolon [2], while Gajbhiye et al. [3] documented a classic complete BD, emphasizing the confusion with diverticula and the importance of intraoperative confirmation. Similarly, Awasthi et al. [4] and Singh et al. [5] highlighted cases with incomplete duplications, some initially mistaken for mesenteric cysts. The rarity of these cases, particularly in adults, and their diagnostic complexity underscores the importance of maintaining a high index of suspicion when interpreting atypical bladder imaging findings.

From an embryological perspective, BD is believed to result from errors during early cloacal development, particularly between the 4th and 7th weeks of gestation. Normally, the cloaca divides into urogenital and anorectal components through formation of the urorectal septum. Disruption or abnormal septation at this stage—such as excessive constriction between urogenital and vesicourethral regions or presence of an accessory cloacal septum—may result in complete or partial duplication of the bladder[7]. The endoderm-derived epithelial lining of the bladder and the mesodermal contributions from the mesonephric ducts (which form the trigone and surrounding musculature) explain the dual embryologic origin and potential for concurrent anomalies in related systems.

**Fig. 8:**
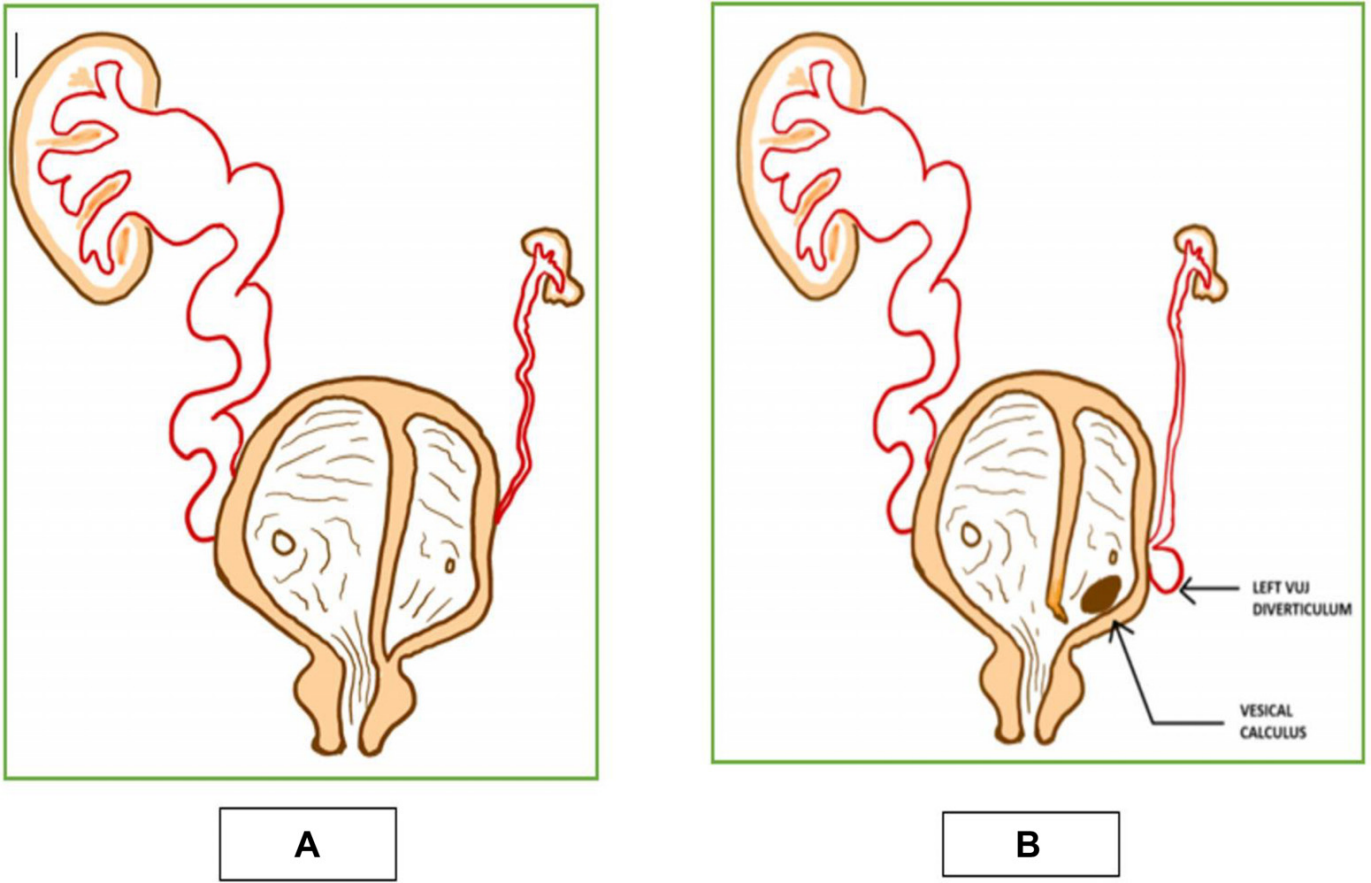
(A) Pictorial representation of complete bladder duplication with a sagittal septum showing divided bladder cavities. The left bladder compartment not communicating with the urethra (obstructed) and associated with renal abnormality. The right compartment exhibiting vesicoureteric reflux with associated hydroureteronephrosis and draining into the urethra. (B) Pictorial representation of current case showing variant of of complete bladder duplication with a sagittal septum. There is a demonstration of small communication between the 2 bladder cavities at the neck of bladder as seen in our case.
• **Incomplete duplication**, where the bladder is partially divided by a septum or membrane, with both chambers draining via a single urethra. This can lead to urinary flow disturbances or incontinence.

**Bladder duplication is classified into 2 major types**:

• **Complete duplication**, where two distinct compartments may have either separate urethras or a single draining urethra (in some cases, one chamber may lack a drainage route altogether, often leading to renal hypoplasia on the affected side) (Fig. 8A).

In the sagittal variant, the division is front-to-back (right and left compartments), while in coronal duplication, the split occurs side-to-side. Sagittal duplication is more common and often seen in females, with a reported female-to-male ratio of 2.5:1[8].

In our case, a complete sagittal septum formed two cavities with a small inter-compartmental communication and a single urethra. The left compartment and left kidney were hypoplastic (Fig. 8B).

Given the bladder’s shared embryologic origin with the genitourinary and lower gastrointestinal systems, BD is frequently associated with other congenital anomalies. These include renal hypoplasia or dysplasia, genital tract malformations (eg, hypoplastic seminal vesicles or prostate), spinal defects (eg, sacral agenesis), and anorectal malformations (eg, imperforate anus) [6]. This is further reflected in associations with VACTERL spectrum anomalies (Vertebral, Anal, Cardiac, Tracheoesophageal, Renal, Limb anomalies). For example, Ramirez-Amoros et al. described duplication associated with a persistent urogenital sinus, further confirming the caudal embryological association [9]. In our case, the concurrent findings of hypoplastic prostate, seminal vesicles, partial sacral agenesis, caudal regression, and history of imperforate anus without prior documentation make this a particularly rare and complex presentation within the spectrum, strengthening the case's significance in literature.

Imaging plays a central role in diagnosis and surgical planning. Ultrasound is typically the first-line modality but may not adequately define internal bladder architecture. CT urography offers detailed anatomical delineation and is particularly useful for identifying associated renal or pelvic anomalies. However, MRI is superior in soft tissue contrast and is considered the most valuable tool for confirming BD, especially in complex or equivocal cases. Gajbhiye et al. [3] and Singh et al. [5] emphasized MRI’s role in differentiating BD from diverticula and mesenteric cysts, conditions that can present with similar clinical features. In our case, a combination of ultrasound, CT, and MRI provided comprehensive anatomical assessment, with definitive diagnosis achieved intraoperatively following multidisciplinary discussion.

If left undiagnosed or untreated, BD can lead to a variety of complications, including recurrent urinary tract infections, urinary retention, incontinence, stone formation (cystolithiasis), and even renal dysfunction, particularly if 1 compartment lacks proper drainage. Treatment is not always necessary and is highly individualized, depending on symptoms, drainage patterns, and renal function. Surgical goals include optimizing bladder capacity, preserving renal function, ensuring proper urinary drainage, and preventing infection or stone formation.

In our patient, recurrent abdominal pain, urinary tract infections, and bladder calculi prompted surgical resection of the bladder septum, resulting in symptomatic relief and improved urinary function.

## Patient consent

Written informed consent for publication of this case was obtained from the patient
